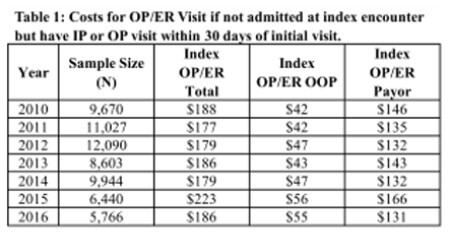# 103 The Long-Term Economic Burden of Burn Injury

**DOI:** 10.1093/jbcr/irae036.102

**Published:** 2024-04-17

**Authors:** Jacob M Dougherty, Hannan A Maqsood, Zhaohui Fan, Stewart C Wang, Mark R Hemmila, Naveen F Sangji

**Affiliations:** Wayne State University School of Medicine, University of Michigan, Pymouth, Michigan; University of Michigan, Ann Arbor, Michigan; Wayne State University School of Medicine, University of Michigan, Pymouth, Michigan; University of Michigan, Ann Arbor, Michigan; Wayne State University School of Medicine, University of Michigan, Pymouth, Michigan; University of Michigan, Ann Arbor, Michigan; Wayne State University School of Medicine, University of Michigan, Pymouth, Michigan; University of Michigan, Ann Arbor, Michigan; Wayne State University School of Medicine, University of Michigan, Pymouth, Michigan; University of Michigan, Ann Arbor, Michigan; Wayne State University School of Medicine, University of Michigan, Pymouth, Michigan; University of Michigan, Ann Arbor, Michigan

## Abstract

**Introduction:**

The long-term economic burden of burn injury on patients and payors for outpatient burn care has not been previously described. For small burns deemed appropriate for outpatient treatment alone, costs can still be significant. While initial hospitalization and intensive care is not required, outpatient burn care may require regular wound care, debridement, skin graft operations, eventual hospital admission, laser treatments, and follow-up appointments placing a large burden on payors and patients. We investigated index and post-acute payor and out-of-pocket (OOP) costs related to burn injury for outpatient care at 30 days, 12 months, 24 months, and 36 months following initial injury to better understand the long-term economic burdens for patients treated for burns without inpatient admission at the time of initial injury.

**Methods:**

An observational cohort study was conducted using a commercial claims database from IBM Watson Health® Marketscan. Patients age ≤ 65 years with an ICD9/10 diagnosis code of burn injury that did not initially require inpatient treatment between 2010 and 2016 were identified and tracked for a three-year period following injury. This cohort was used to determine the payor and OOP costs for burn care during the initial treatment and three-year period following their index encounter through 2019.

**Results:**

We identified 63,540 patients who were treated in an outpatient capacity following their index visitation between 2010 and 2016 (Table 1). Of this cohort, 95.8% received only outpatient treatment while 4.2% received inpatient care within 30 days of their initial visit. The mean inflation-adjusted index out-patient evaluation or emergency room costs were $186 during the study period (Table 1). The mean incremental payor costs for burn care were $2,896 at 30 days, $237 at 12-months, $31 at 24-months, and $15 at 36 months (Table 2). The mean incremental OOP costs for burn care were $588 at 30 days, $27 at 12-months, $4 at 24-months, and $2 at 36 months post-injury for each year from 2010 to 2016 (Table 2).

**Conclusions:**

Burn injury creates significant financial burdens even when the initial injury does not require admission at presentation. This economic burden is highly impactful to both patient and providers, and further investigation of the economic considerations regarding burn injury is an area of interest in burn care.

**Applicability of Research to Practice:**

Allows for a burn team to be cognizant of patient costs associated with ongoing treatment and to provide social work and other resources to patients.